# A Graphene Field-Effect Transistor-Based Biosensor Platform for the Electrochemical Profiling of Amino Acids

**DOI:** 10.3390/bios16020083

**Published:** 2026-01-29

**Authors:** Roanne Deanne Aves, Janwa El-Maiss, Divya Balakrishnan, Naveen Kumar, Mafalda Abrantes, Jérôme Borme, Vihar Georgiev, Pedro Alpuim, César Pascual García

**Affiliations:** 1 Luxembourg Institute of Science and Technology (LIST), 28 Avenue des Hauts-Fourneaux, L-4362 Esch-sur-Alzette, Luxembourg; roanne.aves@list.lu (R.D.A.); janwa.elmaiss@list.lu (J.E.-M.); divya.balakrishnan@list.lu (D.B.); 2 Faculty of Science, Technology, and Medicine, University of Luxembourg, 2 Place de l’Université, L-4365 Esch-sur-Alzette, Luxembourg; 3 DeepNano Group, James Watt School of Engineering, University of Glasgow, University Avenue, Glasgow G12 8QQ, UK; naveen.kumar@glasgow.ac.uk (N.K.); vihar.georgiev@glasgow.ac.uk (V.G.); 4 International Iberian Nanotechnology Laboratory (INL), Avenida Mestre José Veiga s/n, 4715-330 Braga, Portugal; mafalda.abrantes@inl.int (M.A.); jerome.borme@inl.int (J.B.); pedro.alpuim@inl.int (P.A.); 5 Center of Physics of the Universities of Minho and Porto, University of Minho, 4710-057 Braga, Portugal

**Keywords:** amino acids, graphene biosensor, GFET, peptide synthesis, protein sequencing

## Abstract

In this work, we present the introductory methodology for a graphene field-effect transistor (GFET)-based platform for probing the electrochemical fingerprints of amino acids, designed to enable stable and controlled surface chemistry and electrochemical measurements toward peptide and protein sequencing. We begin with a focused conceptual review that motivates electrochemical fingerprinting as a strategy for amino acid and peptide identification and contextualizes this approach within recent advances in protein manipulation relevant to sequencing. We then describe a graphene functionalization protocol that facilitates the directional attachment of amino acids onto the graphene surface. This surface chemistry is quantitatively characterized through surface plasmon resonance (SPR), yielding surface densities in the order of 10^12^ molecules/cm^2^. The same functionalization protocol enables in situ peptide synthesis directly on graphene, as demonstrated by the successful synthesis of a model tripeptide. To support electrochemical interrogation, we developed three complementary platforms for sensor preconditioning, surface functionalization, and titration-based electrochemical measurements, compatible with both aqueous and organic solutions. Preliminary stability measurements indicate a Dirac point drift below 10 mV over 45 min. Altogether, this work establishes the experimental foundations for electrochemical amino acid and peptide fingerprinting using GFET sensors and provides a framework for the future development of electrochemically enabled protein sequencing technologies.

## 1. Introduction

Understanding how molecular alterations affect protein function is crucial for the development of new diagnostic and therapeutic methods. Protein structure and function are primarily determined by the amino acid sequence but can be further modulated by post-translational modifications (PTMs). While a significant part of our knowledge of protein sequences is inferred from genomic data, PTMs and translational errors can only be captured through direct protein sequencing. Despite major advances in genomics, proteomic technologies still lag behind in scalability, throughput, and resolution. This leaves a substantial fraction of biologically relevant proteins and proteoforms uncharacterized, often called the proteomic dark matter, which demand better protein sequencing methods.

Mass spectrometry (MS) is the gold standard for protein analysis, determining the amino acid composition and associated PTMs of peptide fragments based on their mass-to-charge (*m*/*z*) ratios [1,2]. However, de novo sequencing by MS faces limitations in distinguishing isobaric peptides that differ in sequence or PTM positions, as well as in minimizing fragment losses during chemical or enzymatic proteolysis. Furthermore, MS often requires relatively large amounts of purified samples to reconstruct the original protein sequence prior to its digestion. These challenges highlight the need for alternative protein sequencing technologies capable of achieving amino acid-level resolution with improved sensitivity, reduced sample requirements, and potential for multiplexing.

In this article, we present a study of the methods that enable a novel electrochemical approach for amino acid identification that addresses key limitations of existing protein sequencing technologies. We first provide a focused, purpose-driven review of recent advances in next-generation protein sequencing techniques to highlight a critical unresolved challenge: the lack of direct strategies for discriminating individual amino acid fingerprints. The review also presents emerging techniques that enable short peptide manipulation and residue-by-residue cleavage, which could inform design requirements for compact and scalable peptide processing systems that could be used in combination with electrochemical amino acid recognition.

Building on these, we introduce our graphene field-effect transistor (GFET)-based approach that exploits the amphoteric properties of amino acids to resolve unique fingerprints. We describe the associated chemical and electrochemical components required for signal transduction. We then report proof-of-concept amino acid and peptide immobilization and recognition on graphene surfaces. Together, these results illustrate how elements from existing sequencing and sensing strategies can be combined into a novel, simple, and scalable method for electrochemical amino acid profiling using GFETs, laying the groundwork for a future alternative protein sequencing approach.

## 2. Emerging Next-Generation Protein Sequencing Technologies

Protein sequencing currently involves three fundamental steps: (i) proteolysis, (ii) identification of the amino acids and their associated PTMs, and (iii) reconstruction of their correct order along the polypeptide chain. While proteolysis simplifies analysis, it can result in incomplete digestion and the loss of peptide fragments, motivating developments toward sequencing strategies that operate at the single-residue level.

To address single amino acid resolution and small sample quantities, a variety of next-generation protein sequencing concepts has emerged [3]. For these new techniques, single amino acid identification remains a challenge due to the chemical similarity among amino acids, while the structural complexity of intact proteins further complicates whole protein sequencing.

In the following subsections, we discuss the principles and recent developments in these technologies, with a focus on how amino acid identity is accessed and encoded. We highlight the remaining barriers in direct amino acid identification that motivate alternative sensing strategies based on intrinsic amino acid properties, which form the basis of the electrochemical approach introduced in this work.

### 2.1. Nanopore-Based Approaches

Nanopores are nanometer-sized holes in membranes in which the passage of biomolecules disrupts ion flow, resulting in changes in current corresponding to the analyte’s physicochemical properties. While nanopore sequencing has revolutionized nucleotide analysis, adapting this technology to proteins requires solving two main problems: (i) facilitating the translocation of folded, heterogeneously charged proteins and (ii) reliably discriminating among the 20 chemically similar amino acids and their PTMs [4].

Biological nanopores, including α-hemolysin (α-HL), aerolysin, and Curlin sigma S-dependent growth subunit G (CsgG), have been used for protein sequencing due to their defined pore geometries and the ability to control analyte movement through engineered mutations. Controlling protein translocation through the pore is essential to ensure that each amino acid residue dwells in the pore long enough to be read. Multi-pass readings has been shown to improve signal accuracy [5]. Ouldali et al. used aerolysin nanopores and attached a peptide consisting of seven arginine residues to the C-terminal of each amino acid [6]. This gives the peptide an overall positive charge and facilitates electrophoretic translocation through the pore. Zhang et al. used a cucurbit{7}uril (CB7) cavity with a α-HL nanopore [7]. The CB7 cavity interacts selectively with amino acid sidechains within a FGGC(X)D8 carrier peptide, producing distinguishable current blockades for each residue. Through this method, they were able to identify nine amino acids directly and extended this to all 20 amino acids by introducing mutations into the pores and adjusting pH conditions.

Despite progress, reliably distinguishing all amino acids remains a major challenge. Strategies such as attaching charged peptide tags, introducing engineered pore mutations, or using machine learning to deconvolve signals have improved discrimination, but a general solution for direct, residue-level identification of every amino acid has yet to be realized.

### 2.2. Amino Acid Conversion into DNA Barcodes (ProtSeq)

ProtSeq leverages libraries of partially selective peptide or aptamer binders whose interactions with amino acids depend on the amino acid’s physicochemical properties, including charge, hydrophobicity, and size, rather than molecular specificity. The resulting binding patterns are translated into DNA barcodes that can be read using existing genomic next-generation sequencing (NGS) platforms [8]. The binding signatures are computationally deconvolved to infer amino acid identities.

To immobilize the peptides to be sequenced, they are first linked to known oligonucleotides with a unique barcode identifier, creating a peptide-oligonucleotide conjugate (POC). Part of the oligonucleotide sequence is complementary to adapters on a genomic NGS flow cell, fixing the peptides with their N-terminal exposed. Barcode Cycle Sequencing (BCS) begins with the introduction of a library of binders, each consisting of a chain of peptide or aptamer, a linker, and a DNA barcode. Upon binding to the N-terminal amino acid, the binder’s DNA barcode hybridizes with the POC using a ligase, tagging the amino acid with the barcode. The N-terminal amino acid can then be cleaved off and BCS is repeated until a DNA chain that encodes the peptide sequence is generated, which can then be read by standard NGS.

While ProtSeq has successfully demonstrated peptide-to-DNA conversion, peptide sequencing remains to be achieved. The approach faces several challenges, including reliance on large aptamer libraries, long, multi-step workflows, and incompatibility between amino acid cleavage chemistries and DNA barcodes. Importantly, amino acid identity in ProtSeq is inferred from binder interaction patterns which limits the method’s suitability for label-free sensing or minimal chemistry platforms.

### 2.3. Fluorescence-Based Sequencing on a Semiconductor Chip

Another label-dependent strategy for peptide analysis uses fluorescence-based detection on a semiconductor chip. This platform enables single-molecule, real-time peptide sequencing by using fluorescently labeled molecular recognizers [9]. Peptide fragments digested from protein samples are immobilized on the chip via a C-terminal linker. The recognizers bind transiently to the exposed N-terminal amino acid, and the recognition event results in fluorescence with characteristic lifetime, intensity, and kinetic profile. Small, densely packed integrated photodiodes detect the fluorescence signals across the chip surface. Upon dissociation of the recognizer, aminopeptidases cleave the N-terminal amino acid, exposing the next amino acid for subsequent binding and detection. This cycle is repeated until the entire peptide is sequenced, which takes approximately 10 h.

The current range of recognizers does not distinguish between the 20 standard amino acids individually; instead, it groups them into subsets based on similar physicochemical properties. As a result, the platform generates peptide kinetic signature plots, rather than amino acid sequences, that machine learning algorithms analyze to infer residue identity and match peptides against known protein databases.

### 2.4. Edman Degradation on a Microfluidic Chip

Edman degradation, a classical, stepwise method for sequencing peptides by sequentially cleaving and identifying N-terminal amino acids, has remained widely used since its introduction [10], primarily for peptides less than 50 amino acids in length. The process is based on phenylisothiocyanate (PITC) reacting with the N-terminal amino acid under mildly alkaline conditions to form a phenylthiocarbamyl (PTC) derivative. Upon the addition of trifluoroacetic acid (TFA), the N-terminal residue is cleaved off as an anilinothiazolinone (ATZ) intermediate, which subsequently cyclizes into the stable phenylthiohydantoin (PTH) derivative. The released PTH-amino acid is then identified, traditionally by chromatography, and, in modern workflows, through MS.

Building on the foundational principles of Edman degradation, recent advances have miniaturized the chemistry using microfluidic platforms. One implementation integrated nanoliter-scale Edman reactions with Matrix-Assisted Laser Desorption/Ionization-Time-of-Flight (MALDI-TOF)-MS to enable highly sensitive peptide sequencing [11]. The system uses a reaction cartridge packed with C18 particles, serving as a column that retains peptides and minimizes sample loss throughout the sequencing process. The cartridge is sequentially exposed to PITC and trimethylamine to maintain alkaline conditions for efficient coupling, followed by TFA for cleavage. The PTH byproducts are washed away, and the truncated peptides are eluted directly for MALDI-TOF-MS analysis. This subtractive Edman degradation workflow identifies each residue from the mass shift of the remaining truncated peptide.

Performance tests demonstrated approximately 80% peptide retention after each Edman cycle, including across proline residues, which typically hinder classical Edman chemistry. This high retention enables sequencing from as little as 0.2 fmol of sample. These advances highlight that controlled N-terminal cleavage chemistry is still a powerful tool for accessing residues sequentially; however, identification still relies on MS rather than direct readout of the released amino acids.

### 2.5. Fluorosequencing

Fluorosequencing relies on selectively labeling amino acids in peptide fragments from a single molecule before immobilizing them on a glass surface [12]. So far, cysteine, lysine, phosphorylated serine, and phosphorylated lysine residues have been successfully labeled with fluorophores. Total internal reflection fluorescence (TIRF) microscopy is then used to excite the fluorophores and capture images of the peptides as they undergo cycles of Edman degradation. A stepwise decrease in fluorescence intensity is observed as labelled amino acids are cleaved, generating sequence-specific fluorescence decay patterns that serve as peptide fingerprints. This technique enables highly parallelized analysis of zeptomole quantities, approaching single-molecule sensitivity, and has been demonstrated to analyze millions of peptides within a small surface area.

Despite the high throughput, fluorosequencing only provides intermittent sequence information, since fluorescent labeling is limited to a few amino acids and their modifications. As a result, it generates sparse peptide fingerprints that require computational mapping to protein databases rather than enabling direct identification per residue.

### 2.6. Base-Induced N-Terminal Cleavage

Like Edman degradation, alkaline-based peptide sequencing relies on derivatizing the N-terminal amino acid so that residues can be sequentially cleaved and identified. Classical Edman chemistry, while widely used, has some notable limitations: sequencing often stalls at proline (especially at position 2), the PTH derivative of tryptophan is unstable, and cysteine requires prior oxidation or alkylation. These challenges, along with its incompatibility with fluorosequencing approaches, have prompted the development of alternative N-terminal chemistries.

A recently reported alkaline-based N-terminal sequencing methodology offers an alternative that overcomes these limitations [13]. Instead of isothiocyanate, the method uses DR3, which has an N-hydroxysuccinimide leaving group linked to a hydrazinecarboxamide nucleophile to derivatize the N-terminal amino acid. DR3 effectively derivatized all 20 amino acids at the N-terminus, with cysteine requiring an iodoacetamide cap to improve conversion. The reported conversion efficiencies ranged from 60% to 93%. Sequential cleavage of up to three amino acids has been achieved, and parallelization was demonstrated with a derivatization efficiency of 84% in a mixture of 50 unique peptides from digested bovine serum albumin.

Compared with classical Edman degradation, this alkaline-based strategy offers several advantages. It is performed under milder conditions, which reduces the degradation of sensitive residues. The workflow is simplified because cleavage and precipitation can be performed without additional purification steps. Importantly, unlike thiocyanate-based derivatization, this approach preserves the physicochemical properties of the N-terminal amino acid, maintaining its native charge and amphoteric behavior. This preservation provides a chemical foundation for strategies that exploit intrinsic residue properties, including downstream physicochemical or electrochemical sensing approaches. By retaining the inherent characteristics of the amino acids, the method establishes a pathway for residue-level identification that does not rely on labels, directly informing the design of novel electrochemical amino acid profiling platforms.

### 2.7. Summary and Perspectives

Despite substantial progress, most protein sequencing approaches still rely on pattern matching, where experimental data are compared to reference databases constructed from translated DNA sequences. While effective for known proteins, this strategy cannot provide true de novo sequence information for unknown proteins or novel proteoforms. Advances in controlled enzymatic proteolysis, as well as the alkaline degradation of N-terminal peptides, have begun to enable more direct determination of amino acid order. However, current methods still face challenges in reliably identifying amino acids and their PTMs. In the following sections, we present an alternative sensing strategy that targets this gap by transducing intrinsic amino acid fingerprints and describe the first steps toward its implementation.

## 3. Electrochemical Readout of Amino Acid Amphoterism for Protein Sequencing

**Figure 1:**
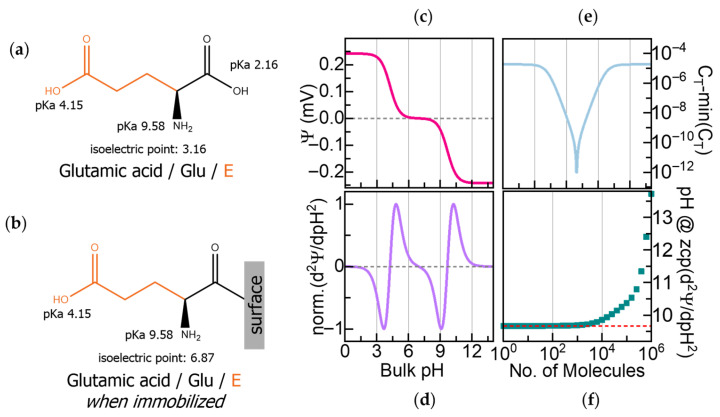
(**a**) Glutamic acid (Glu) with its acidity constants (pKa) and isoelectric point (pI). (**b**) The pI of glutamic acid changes when it is immobilized through its C-terminal due to the deactivation of that pKa. (**c**) Calculated surface potential (Ψ) of 100 molecules/μm^2^ of C-terminal immobilized Glu on a field-effect transistor (FET) as a function of pH. (**d**) Second derivative of Ψ (δ^2^Ψ/δpH^2^) showing zero crossover points (ZCPs) corresponding to the pKa and pI in (**b**). (**e**) Calculated capacitance (C_T_) during Glu titration. (**f**) Influence of surface density on ZCP accuracy.

In our proposal, we leverage the amphoteric nature of amino acids for molecular discrimination. As the concentration of H^+^ ions changes, the net charge of each amino acid varies, and they exhibit a behavior defined by the acidity constants (pKa) of their C-terminal, N-terminal, and sidechain functional groups [14]. This relationship between net charge and pH can generate a characteristic electrochemical fingerprint for each amino acid. To illustrate the method, we consider glutamic acid (Glu) which displays marked pH-dependent electrochemical behavior arising from its multiple ionizable groups. It has an N-terminal with a pKa of 9.58, a C-terminal with a pKa of 2.16, and a carboxylic acid sidechain with a pKa of 4.15, resulting in an isoelectric point (pI) of 3.16 (Figure 1a). Below pH 2.16, Glu is fully protonated with a net charge of +1, between pH 2.16 and 4.15, deprotonation of the C-terminal yields an overall neutral charge, above pH 4.15 the sidechain is deprotonated resulting in a negative charge, and above pH 9.58, Glu reaches a −2 charge when its N-terminal is deprotonated. Following pH changes during titration with a strong base is a way to identify amino acids [15]. Near the different pKa values, the amino acids’ buffering effects are observed as plateaus in the pH vs. [OH^−^] plots [16]. However, traditional titration requires molar equivalents of the amino acid analyte and the base, which would not be practical for modern sequencing purposes.

To identify the components of a few peptide fragments, we propose sequentially immobilizing the extracted amino acids onto FET sensors and transducing their pH-dependent charge variations by recording changes in surface potential and capacitance. Our previous work using a computational model report that immobilized amino acids, even those with similar masses, exhibit distinct electrochemical fingerprints arising from characteristic shifts in surface potential and capacitance across pH [17].

We can illustrate the fingerprint determination on the surface potential and capacitance with Glu, whose intrinsic amphoteric properties have been described above. Immobilizing Glu on a sensor surface through its C-terminal deactivates its pKa at pH 2.16 and shifts the original pI of the molecule to pH 6.87, as shown in Figure 1b. Figure 1c shows the calculated pH-dependent surface potential (Ψ) for 100 molecules/μm^2^ of C-terminal immobilized Glu. The induced surface potential is positive in the acidic range below its pI, negative above its pI, and zero at its zwitterionic state at its pI. Furthermore, the second derivative of the surface potential (δ^2^Ψ/δpH^2^), presented in Figure 1d, reveals the electrochemical signature of Glu with three zero crossover points (ZCPs) that correspond to Glu’s two active pKa values at pH 4.15 and pH 9.58 and the immobilization-shifted pI at pH 6.87.

The total capacitance (C_T_) depends on both the dielectric constant and the effective thickness of the dielectric layer, which are determined by the molecular charges and the length of the immobilized amino acid, providing an orthogonal signal to the surface potential. Since capacitance is inversely proportional to dielectric thickness, shorter amino acids yield higher C_T_ values, whereas longer or bulkier residues will produce lower C_T_ values. Figure 1e shows the calculated C_T_ as a function of pH, reaching its highest value when the molecule is fully charged and its lowest at the neutral state. While the pH-dependent variation of C_T_ due to charging is symmetric to the surface potential, its minimum at the neutral state reflects the effective molecular length and dielectric properties, enabling discrimination between residues based on their physical structure.

Our computational model also revealed a saturation effect at high surface densities of immobilized molecules. As shown in Figure 1f, the ZCP values deviate from their expected positions when molecular coverage exceeds 1 × 10^14^ molecules/μm^2^, underscoring the importance of controlling surface density. The model accounts for proton affinities, dielectric constants, molecular lengths, and surface density. These findings guided the design of our workflow.

Both N-terminal and C-terminal immobilization are, in principle, compatible with electrochemical fingerprinting and will yield distinct signatures. In our previous work, we have also reported N-terminal immobilization and its associated calculated electrochemical responses [17]. In the present work, C-terminal immobilization is intentionally chosen to be compatible with N-terminal cleavage strategies. This configuration also reflects the natural N-to-C reading direction for proteins.

While Glu is used here as a representative example, the same principle is extendable to the other 19 standard amino acids, as their characteristic amphoteric behavior and varying sidechains produce distinct electrochemical fingerprints. The calculated responses for the other amino acids, along with some short peptide sequences, are reported in our previous work [17], illustrating the general applicability of this approach.

We propose using GFETs to detect charge variations associated with amino acids. Graphene, a two-dimensional hexagonal lattice of carbon atoms, offers high carrier mobility and chemical stability, making it an effective transducer for biosensing. The key requirement of this approach is the selective transduction of amphoteric charge transitions originating from amino acids and peptides. Unlike conventional oxide-based FET sensors and other alternative FET platforms, graphene lacks hydroxyl groups that interact with hydrogen ions, thereby minimizing parasitic amphoteric effects and reducing signal interference. Consequently, pH-dependent surface potential variations measured on graphene arise predominantly from the protonation and deprotonation equilibria of the immobilized amino acids and peptides, rather than from the transducer itself.

As a truly two-dimensional material, graphene enables direct coupling of probe molecules to its surface without an intervening dielectric layer, maximizing capacitive coupling. Moreover, graphene exhibits a unique electronic band structure characterized by the Dirac point—a distinct conductance minimum that is highly sensitive to surface charge perturbations. Interactions between biomolecules and graphene induce measurable shifts in the Dirac point that are directly proportional to changes in surface potential, making GFETs particularly well suited for resolving amphoteric charge fingerprints.

Here, we focus on optimizing a methodology for measuring surface potential changes, offering a faster and more direct readout than capacitance measurements. Unlike capacitance, which requires frequency-dependent analysis or optimization at a single operating frequency, surface potential can be tracked in real time. We first describe the surface functionalization and amino acid immobilization, followed by an analysis of GFET sensor properties to evaluate the feasibility of our approach.

## 4. Materials and Methods

### 4.1. Chemicals

Dimethyl sulfoxide (DMSO), 1-pyrenemethylamine (PMA), phosphate-buffered saline (PBS) tablets, acetonitrile (anhydrous), N,N-diisopropylethylamine (DIPEA), TFA, acetic anhydride, 1-dodecanethiol (DDT), potassium hydroxide (KOH), and potassium nitrate (KNO_3_) were purchased from Merck (Darmstadt, Germany). N-(tert-Butoxycarbonyl)-L-glutamic acid 9-fluorenylmethyl ester (Boc-L-Glu(OFm)-OH) and Boc-L-Asp(OFm)-OH were synthesized by Bachem (Bubendorf, Switzerland). Hexafluorophosphate benzotriazole tetramethyl uranium (HBTU) and Nα-(tert-Butoxycarbonyl)-Nε-(9-fluorenylmethoxycarbonyl)-L-lysine (Boc-L-Lys(Fmoc)-OH) were from Iris Biotech (Marktredwitz, Germany).

### 4.2. Surface Plasmon Resonance (SPR) Analysis

SPR measurements were performed using a BioNavis MP-SPR Navi^TM^ 200 OTSO (Tampere, Finland) equipped with a laser operating at 670 nm, a polyether ether ketone (PEEK) prototype injection cell, and a solvent-resistant tubing system. Gold SPR sensor slides were purchased from BioNavis and were sent to General Graphene Corporation (Knoxville, TN, USA) for graphene transfer. The resulting graphene-coated SPR sensor slides will be hereafter referred to as graphene-SPR chips.

Before sample measurements, 0.1%, 0.25%, 0.5%, 1%, 2%, and 5% (*v*/*v*) glycerol-water solutions were used to calibrate the instrument’s optical response with the graphene-SPR chips. The refractive index (RI) values of the glycerol solutions were calculated using a linear combination of the refractive indices of the solution components [18]. The corresponding resonance angle shifts were recorded, and the angular sensitivity factor (S) was determined, representing the change in RI per change in resonance angle (Δn/Δθ). This calibration factor was subsequently used to convert measured resonance angle shifts (Δθ) into RI changes ${(n}_{A}-n_{C})$ according to:

(1) $$n_{A}-n_{C}=S\cdot \;\Delta \theta$$

All SPR measurements were performed in 1× PBS. Resonance angle shifts were analyzed to monitor molecular attachment or removal. The resonance angles were determined by fitting the curves to a Gaussian function using OriginPro 2019b.

### 4.3. Optimization of Graphene Surface Functionalization

A graphene-SPR chip was sequentially incubated in 1 mM, 10 mM, 20 mM, and 30 mM PMA in DMSO overnight in a glass container. After each incubation, the chips were rinsed with DMSO, ethanol, and distilled water, dried under N_2_ flow, and an SPR angular scan was performed in 1× PBS. After the final incubation in 30 mM PMA, an additional 30-min prolonged rinsing step under acetonitrile flow was performed. Based on these optimization experiments, a PMA concentration of 20 mM was selected and was used for all subsequent experiments requiring graphene functionalization.

### 4.4. Amino Acid Coupling and Peptide Synthesis

Amide bond formation between PMA primary amines and the C-terminal of Boc- and sidechain-protected amino acids (10 eq.) was carried out using HBTU (10 eq.) and DIPEA (20 eq.) as activating agents, following our previously reported acid-mediated solid-phase peptide synthesis conditions [19]. Coupling was performed twice for five minutes each. Residual amino groups were quenched for five minutes with 4.5% (*v*/*v*) acetic anhydride, and N-terminal Boc groups were removed with 50% TFA. All reagents were prepared in acetonitrile unless stated otherwise. Each step was followed by rinsing with acetonitrile and drying with air to remove residual reagents from the microfluidic channels and prevent reagent mixing. For peptide synthesis, additional amino acids were sequentially coupled under identical conditions.

All amino acid coupling and peptide synthesis steps were carried out in flow using an Ismatec^®^ Reglo ICC peristaltic pump (Avantor Inc., Radnor, PA, USA) connected to the BioNavis MP-SPR machine holding the graphene-SPR chip. SPR angular scans were taken in 1× PBS after each coupling and deprotection step.

### 4.5. Surface Density Calculation

Surface densities of immobilized molecules were estimated from the RI changes ($n_{A}-n_{C}$) derived from the SPR signal using the De Feijter formula (Equation (2)) [20,21]:

(2) $$\Gamma =\;\frac{d_{A}(n_{A}-n_{C})}{dn/dc}\;$$

where Γ is the surface concentration (g/cm^2^) and $d_{A}$ is the thickness of the adsorbed layer, estimated from the theoretical volume or surface area of the molecule added (from literature or manufacturer data, see Table 1). The RI increment (*dn*/*dc*) values for each molecule, obtained from literature, are also listed in Table 1.

To convert the calculated adsorbed mass into the number of molecules per unit area, Γ was divided by the molecular weight (*MW*) of the immobilized molecule, and then multiplied by the Avogadro constant $N_{A}$:

(3) $$N=\;\;\frac{\Gamma }{MW}\;\times \;N_{A}$$

where *N* is the surface density in molecules/cm^2^.

### 4.6. Graphene Field-Effect Transistor (GFET) Sensors

**Figure 2:**
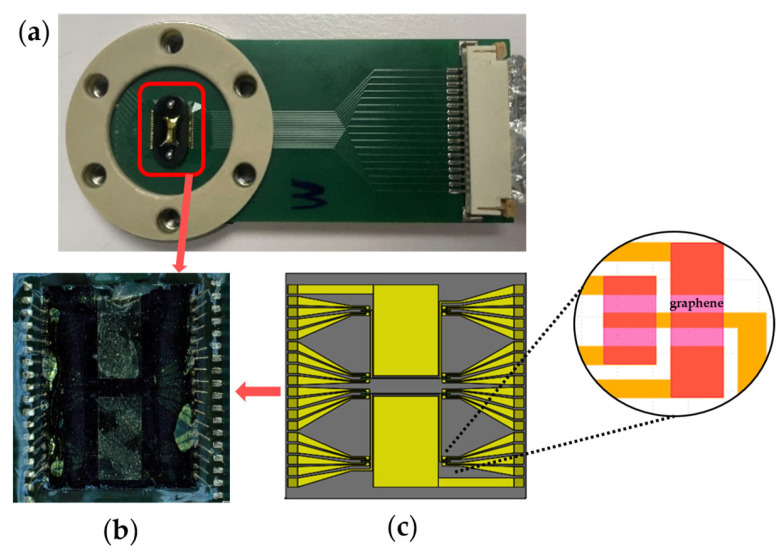
Graphene field-effect transistor (GFET) chip for the electrochemical profiling of amino acids. (**a**) GFET chip integrated on a printed circuit board (PCB) with gold wire bonding for signal transmission and communication with our electronic system. (**b**) Zoomed-in view showing the wirebonded GFET chip. (**c**) Schematic illustration of (**b**) showing the GFET chip’s architecture which contains two coplanar gate electrodes and two source electrodes, each shared by 16 drain electrodes, to form 32 different sensors. (inset) Detailed view of four graphene sensors.

The GFET chips were fabricated using the cleanroom lithography process previously described [25,26]. Each 5 mm × 5 mm GFET chip contains 32 GFET sensors with corresponding drain electrodes; a representative GFET chip is shown in Figure 2b. Sixteen drain electrodes share a common source and a coplanar gold gate electrode, forming the sensor architecture illustrated schematically in Figure 2c. Each GFET chip was screened for sensors with resistance measurements not exceeding 2.5 MΩ on a probe station (Karl Suss MicroTec PM8, SUSS MicroTec, Garching, Germany) before being mounted on a printed circuit board (PCB), as shown in Figure 2a. Electrical contact was established through gold wire bonding, and the bond pads and gold wires were protected with polydimethylsiloxane (PDMS, Sylgard™ 184 Silicone Elastomer Kit, Dow Chemical Company, Midland, MI, USA).

### 4.7. Custom Sensor-Microfluidic Platforms for GFET Workflow

**Figure 3:**
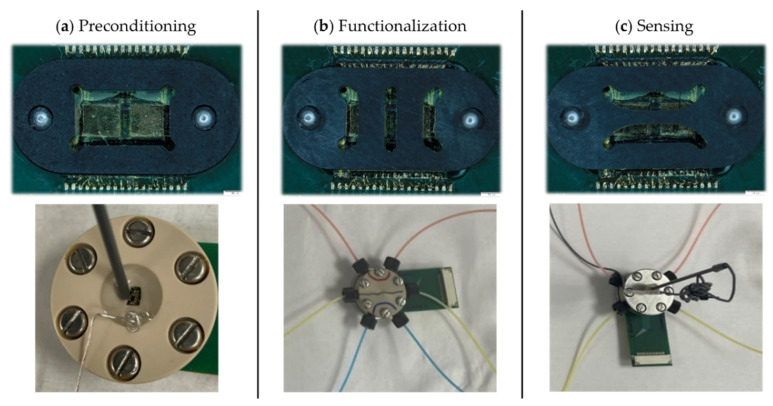
Custom sensor-microfluidic platforms developed for the different phases of the GFET workflow. (**a**) Preconditioning platform with a single large opening for the electrochemical cleaning and passivation of the coplanar gate electrodes. (**b**) Functionalization platform for implementing the SPR-optimized protocols on GFET sensors, with three independent vertical microfluidic channels. (**c**) Sensing platform for aqueous titration with two independent horizontal microfluidic channels; one channel incorporates an external Ag/AgCl reference electrode. The (**top row**) shows the gaskets unique to each platform and the (**bottom row**) shows the corresponding platforms in their mounted configurations. Colored liquids were used in the functionalization and sensing platforms to illustrate the independence of the channels.

Three custom prototype platforms were developed to hold the PCB-mounted GFET chips through the different experimental phases: (a) preconditioning, (b) functionalization, and (c) sensing (Figure 3). Each platform was designed based on the specific chemical, electrical, and mechanical requirements of its respective experimental steps and features a unique chemically resistant perfluoroelastomer (FFKM) gasket. The gaskets enable microfluidic integration and confine reagents to defined areas of the chip, protecting the chip from delamination during prolonged exposure to harsh chemicals.

The gasket in the preconditioning platform (Figure 3a) defines a single large opening that exposes the coplanar gate electrodes for electrochemical cleaning and passivation. Electrochemical cleaning was performed through cyclic voltammetry in 10 mM H_2_SO_4_, using the gate electrode as the working electrode, a platinum wire as the counter electrode, and an Ag/AgCl electrode as the reference electrode. Directly after, the gate electrodes were passivated by incubation in 2 mM DDT in ethanol for four hours, then rinsed with ethanol and distilled water and dried under N_2_ flow. These preconditioning steps ensure a clean, consistent starting condition for all devices.

The functionalization platform (Figure 3b) is engineered to translate the optimized functionalization and peptide synthesis protocols onto the GFET chips. This platform is made with PEEK to withstand harsh organic solvents. The gasket defines three independent vertical microfluidic channels, allowing three different surface chemistries to be performed on a single GFET chip. Functionalization with linker molecules, amino acid coupling, and peptide synthesis are performed in this platform, each followed by rinsing with acetonitrile and water and drying under N_2_ flow. The microfluidic flow system enables uniform, controlled flow rates across all three channels, maximizing reaction efficiency and ensuring reproducible surface functionalization.

The sensing platform, also fabricated in PEEK, is used for the electrochemical measurement of the GFET sensors, while the microfluidic flow enables inline titration. Its gasket defines two horizontal microfluidic channels, one of which accommodates an external wired Ag/AgCl reference electrode (Dri-Ref™-2, World Precision Instruments, Sarasota, FL, USA; Figure 3c). This configuration allows direct comparison of the same surface functionalization under two different reference electrodes: the coplanar gate electrode and the external reference electrode, enabling evaluation of gating stability and noise performance for precise Dirac point determination. pH titrations are performed and monitored externally with a pH meter before the solution is introduced through the microfluidic channels to ensure control of solution conditions during measurements.

The flow conditions were optimized for the functionalization and sensing platforms. The three vertical channels of the functionalization platform required a slower flow rate of 42.5 μL/min to maximize reagent interaction with the surface and avoid leakage. The longer horizontal channels of the sensing platform allowed for a higher flow rate of 107 μL/min.

Altogether, these custom sensor-microfluidic platforms minimize human error, reduce variability from manual handling, and ensure the safe, controlled delivery of hazardous reagents throughout the GFET workflow.

### 4.8. GFET Electrochemical Characterization

GFET measurements were performed using a Keithley 2636B System SourceMeter and a Keithley 3706A System Switch/Multimeter (Tektronix, Inc., Beaverton, OR, USA) controlled via a custom LabVIEW program. The system enabled the consecutive measurement of 16 sensors at a time. A constant source-drain voltage (V_SD_) of 10 mV was applied while the gate-source voltage (V_GS_) was swept between −0.8 V and 0.8 V using the coplanar gate electrode or an external Ag/AgCl reference electrode to generate transfer curves (I_SD_ vs. V_GS_). Electrochemical measurements were performed while the platform was inside a Faraday cage to minimize electrical noise. Each V_GS_ sweep collected 91 measurement points per sensor.

The Dirac point was determined from the transfer curves obtained in 1× PBS by fitting the curve to a Gaussian function on OriginPro 2019b. To monitor the Dirac point drift, the V_GS_ sweep was narrowed down to a 300 mV window per GFET sensor, ensuring that the predetermined Dirac point remains centered within this range. The 300 mV V_GS_ sweeps were repeated over 45 min under constant conditions.

### 4.9. pH Titration

A 0.1 M KNO_3_ buffer was titrated with incremental additions of 0.1 M KOH buffer, and the pH was monitored with a calibrated pH meter (XS pH 7 Vio Portable pH Meter, XS Instruments, Carpi, Italy). After the pH has stabilized, transfer curves were generated with an external Ag/AgCl reference electrode for gating.

## 5. Results and Discussion

We present a systematic approach to developing and characterizing a graphene-based platform for amino acid profiling. SPR and electrochemical measurements serve complementary roles. SPR provides an independent means to validate surface chemistry and quantify molecular surface densities, ensuring that the functionalization proceeds as expected. First, we optimized a protocol for functionalizing graphene with a linker molecule to enable robust, reproducible immobilization of amino acids. Then, we showed the synthesis of short peptides on the graphene surface, with layer-by-layer monitoring of surface density.

Electrochemical measurements, on the other hand, are used to probe the electrical response of the GFET sensors once functionalization has been established. To pave the way for reliable sensing, here we evaluated the baseline performance of the GFET sensors by characterizing key electrochemical properties, including Dirac point behavior over time and any residual pH sensitivity from defects, showing potential for amino acid profiling with a few molecules.

### 5.1. Optimization of Graphene Functionalization

To facilitate the measurement of the surface potential of molecules such as amino acids and peptides, graphene requires molecular linkers for their immobilization on its surface. Graphene’s unique electronic properties stem from its sp^2^ lattice structure. While direct covalent immobilization to graphene can be achieved by breaking some carbon bonds, it compromises electrical performance and alters the Dirac point. Noncovalent functionalization is preferred because the adsorption of molecules through π-π interactions preserves the lattice and the intrinsic electronic properties essential for sensing [27]. Pyrene derivatives, such as 1-pyrenebutyric acid N-hydroxysuccinimide ester (PBASE), have been extensively used for this purpose [26,28,29,30]. PBASE features a pyrene moiety that stacks onto graphene and a succinimidyl ester group for biomolecule coupling. However, esters are labile and highly susceptible to hydrolysis in aqueous environments [31], which can compromise subsequent steps. Ester bonds are inherently weak, and exposure to harsh reagents used for amino acid immobilization and peptide synthesis, as well as to acidic and basic pH conditions during titration, poses a significant risk to bond stability [31,32]. Consequently, PBASE was not used as a linker in this work.

**Figure 4:**
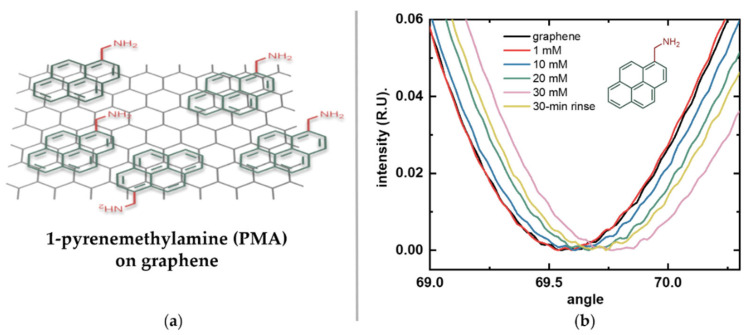
Functionalization of graphene with the linker molecule 1-pyrenemethylamine (PMA). (**a**) Schematic illustration of PMA noncovalently attached to graphene via π-π stacking of its pyrene moiety (green), leaving its terminal amino group (red) exposed for subsequent binding. (**b**) Surface plasmon resonance (SPR) response of bare graphene (black) after sequential incubations with increasing PMA concentrations: 1 mM (red), 10 mM (blue), 20 mM (green), 30 mM (pink), and after prolonged rinsing (yellow).

To enable immobilization of amino acids via their C-terminal, the linker must provide stable amino groups. This allows the formation of strong amide bonds and ensures consistent directional attachment, provided that the N-terminal and sidechains are chemically protected, as in standard peptide synthesis. Based on these considerations, we selected PMA as our linker molecule (Figure 4a). This pyrene derivative has a short methyl spacer that keeps the immobilized amino acids within the Debye length—the characteristic distance over which electrostatic interactions are screened in an electrolyte solution—which is about 0.7 nm in 1× PBS [27]. This ensures efficient transduction under physiological conditions. The short spacer also minimizes steric hindrance while providing a high surface density of reactive amino groups without overcrowding.

Figure 4b shows the reflected light intensity versus incidence angle curves for the graphene-SPR chip after each overnight incubation in increasing PMA concentrations (1 mM, 10 mM, 20 mM, and 30 mM) and after prolonged rinsing. These angular scans show continuous positive shifts in the SPR resonance angle as PMA concentration increased. After incubation in the highest concentration, prolonged rinsing revealed a decrease in signal, suggesting that some of the observed increase was due to non-specific adsorption rather than stable π-π stacking. These results also suggest that stable surface saturation was achieved at a concentration closer to 20 mM. Therefore, 20 mM PMA was selected as the linker concentration for all subsequent graphene functionalization experiments.

Calibration of the SPR instrument with the graphene-SPR chips using glycerol–water solutions yielded an angular sensitivity factor *S* of 0.01163 RIU/deg (R^2^ = 0.997). This value was subsequently used to convert measured resonance angle shifts into RI changes using Equation (1). To determine the number of immobilized PMA molecules, the SPR curves for bare graphene and the final rinse after sequential PMA incubations were analyzed. Using Equations (2) and (3), the surface density of PMA was calculated to be approximately 1.84 × 10^12^ ± 3.67 × 10^10^ molecules/cm^2^. In our previous work, PBASE adsorption was quantified indirectly via DNA conjugation using Quartz Crystal Microbalance with Dissipation (QCM-D) measurements performed on graphene transferred onto quartz crystals. This approach yielded a surface density of 1.27 × 10^13^ molecules/cm^2^ for PBASE, corresponding to an estimated 25% surface density assuming face-to-face pyrene adsorption on graphene [33]. The lower surface density observed here can be attributed to several factors, including the approximation of the *dn*/*dc* value and the presence of residual polymers on the graphene surface resulting from graphene transfer and cleanroom microfabrication, which reduces the effective adsorption area for PMA. Therefore, this reported number represents a conservative estimate of stable PMA coverage. Overall, these results demonstrate effective attachment and sufficient molecular coverage of PMA on graphene, enabling efficient amino acid coupling and reproducible downstream measurements.

### 5.2. Amino Acid Coupling

**Figure 5:**
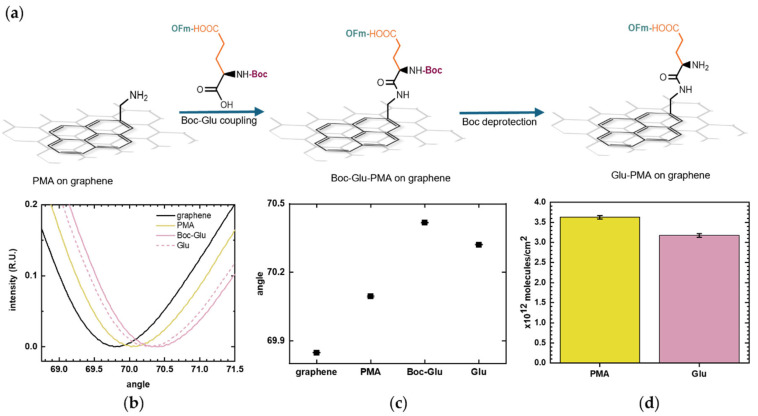
Glutamic acid (Glu) coupling to 1-pyrenemethylamine (PMA). (**a**) Schematic illustration of Boc-L-Glu(OFm)-OH binding to the exposed amino group of PMA through its C-terminal and the subsequent removal of Boc from its N-terminal. (**b**) SPR curves showing the layer-by-layer surface modification: baseline on bare graphene (black), PMA linker (yellow), Boc-L-Glu(OFm)-OH coupling (pink), and Glu deprotection (pink dashed). (**c**) SPR resonance angles of the sequential surface modification. Positive shifts correspond to the addition of molecules on the surface while negative shifts indicate removal of molecules. Error bars represent uncertainties obtained from curve fitting. (**d**) Estimated surface density of PMA and Glu immobilized on the graphene surface calculated using the De Feijter equation. Error bars correspond to the calculated standard errors.

Following PMA linker immobilization, amino groups are available for coupling with amino acids. Glu, with its N-terminal protected with the acid-labile Boc molecule and its sidechain protected with the base-labile OFm molecule, was added to covalently bond to PMA through its C-terminal (Figure 5a), following a previous protocol that we developed for oxide surfaces [19]. The SPR measurements, showing reflected light intensity versus incidence angle for each functionalization step, are shown in Figure 5b. The immobilization of the amino acid onto the PMA-functionalized graphene surface was monitored through the increase in the SPR resonance angle.

To better estimate the number of immobilized Glu molecules, the Boc molecule was removed as it added some molecular mass to the surface, which contributes to the SPR signal. The deprotection was observed as a decrease in the resonance angle, as shown in Figure 5c, which plots the SPR resonance angle corresponding to each surface functionalization step. The number of immobilized Glu was calculated and was compared with the PMA coverage. Figure 5d presents the molecular surface densities calculated from the SPR resonance angles at each stage of the modification process. The number of immobilized Glu molecules should be equal or lower than the number of PMA molecules, as the coupling yield with PMA is inherently less than 100%. The amino acid coupling was shown to be effective, with Glu surface density (3.18 × 10^12^ ± 4.32 × 10^10^ molecules/cm^2^) falling within the same order of magnitude as the PMA linker (3.62 × 10^12^ ± 4.31 × 10^10^ molecules/cm^2^). This graphene-SPR chip exhibited a slightly different surface density compared to that in Figure 4, but the values remain on a comparable scale. Such variation is reasonable and can arise from differences in graphene defect density and local surface cleanliness.

### 5.3. In Situ Peptide Synthesis on Graphene

To modulate the amphoteric behavior of amino acids at the graphene surface, we could use stepwise peptide growth to introduce additional charged side chains. This approach enables controlled tuning of the surface charge by either increasing a specific charge type or alternating between positive and negative charges through sequence design.

As proof-of-concept, the tripeptide Lys-Asp-Lys was synthesized as a model to eventually examine how alternating positively charged (Lys) and negatively charged (Asp) residues influence the response of GFET sensors. Supplementary Figure S1 shows the calculated surface potentials of the model peptides Lys, Lys–Asp, and Lys–Asp–Lys (Supplementary Figure S1). After each coupling step, the successive addition of amino acids is expected to produce pH-dependent shifts in surface potential as the net surface charge alternates between positive and negative.

**Figure 6:**
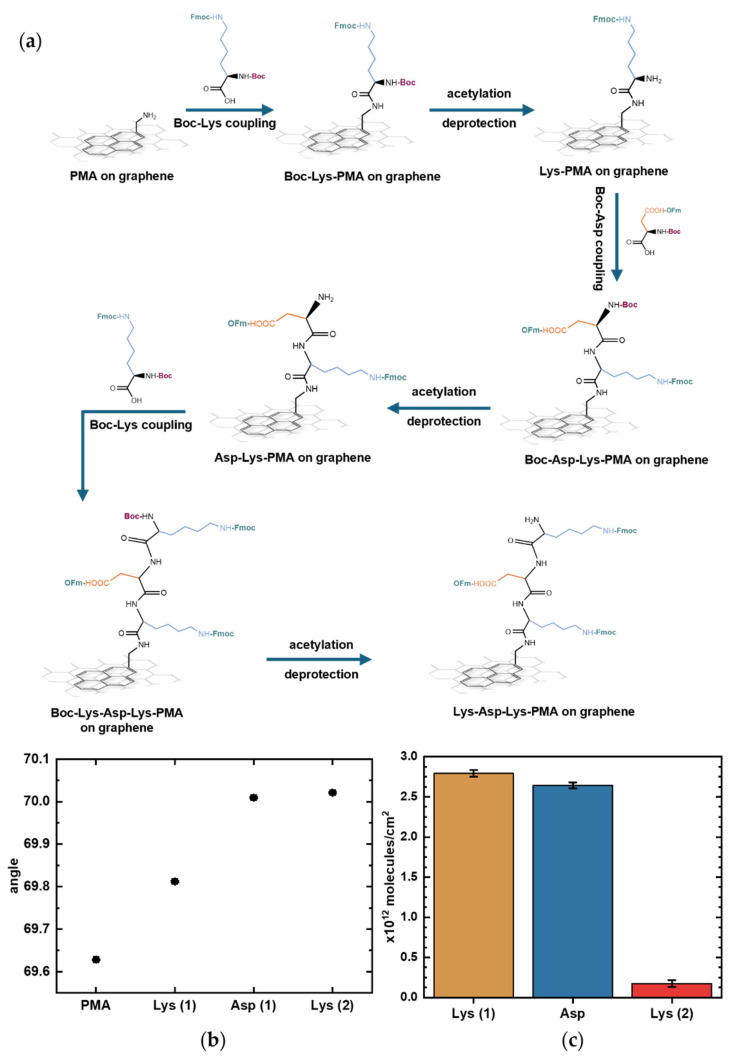
On-graphene synthesis of the representative peptide Lys-Asp-Lys monitored by surface plasmon resonance (SPR). (**a**) Schematic of step-by-step Lys-Asp-Lys chain elongation on graphene with PMA as linker. (**b**) SPR curves showing shifts corresponding to each deprotection step, when only the sidechain-protected amino acid remains immobilized. Error bars represent uncertainties obtained from curve fitting. (**c**) Estimated surface density of Lys (1), Asp, and Lys (2) on the graphene surface as peptide synthesis progressed, calculated using the De Feijter equation. Error bars correspond to the calculated standard errors.

To synthesize peptides, we adapted our previously reported solid-phase peptide synthesis protocol designed for conventional oxide FET devices [19]. Briefly, the method involves removal of the acid-labile Boc group protecting the N-terminal, while base-labile protecting groups are used for the sidechains. We performed cycles of (1) coupling of Boc-protected amino acids with base-labile sidechain protection twice, (2) acetylation to quench any free amino groups, and (3) deprotection of the N-terminal under acidic conditions in preparation for coupling with the next amino acid (Figure 6a). All sidechains were kept protected to ensure the directionality of the synthesis.

SPR measurements showed progression in the resonance angle shifts during peptide elongation on the graphene surface. Figure 6b shows the resonance angles corresponding to each functionalization step; each upward shift marks the deprotection step after amino acid coupling in which only the sidechain-protected amino acid remains bound to the surface. Tracking SPR angle shifts, even during deprotections steps, enables in-process assessment of Boc deprotection and amino acid coupling efficiency. An acetylation step was introduced after each coupling step to quench any unreacted amino groups and prevent unwanted chain elongation. However, because the acetylation step only introduces a few small molecules to block remaining reactive sites, it produced minimal SPR angle shifts that were not reliable enough to be quantifiable. The functional importance of acetylation, despite its negligible SPR angle shift during synthesis, is demonstrated in Supplementary Figure S2, which shows that performing acetylation during peptide synthesis reduces nonspecific antibody binding. The product quality from this synthesis protocol was previously confirmed by MS, showing a 90% yield with the synthesis of a different, longer pentapeptide sequence on a gold SPR chip without graphene [19].

### 5.4. Electrochemical Characterization of the GFET Sensors

**Figure 7:**
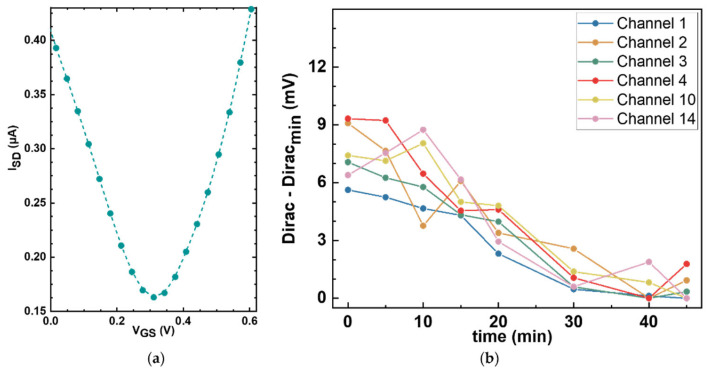
Electrochemical characterization of bare graphene sensors. (**a**) Representative bare graphene transfer curve measured in 1× PBS, showing the Dirac point at around 0.3 V. A 0 V to 0.62 V V_GS_ sweep window was applied. (**b**) Stability assessment of the Dirac point over time. Repeat transfer curve measurements were taken from six different unfunctionalized sensors on a single GFET chip at different time points, and the Dirac points were plotted as a function of time. Minimal drift of less than 10 mV was observed over 45 min.

The next step in building our platform is to characterize the electrical response of the GFET sensors. The electrical performance of each sensor was assessed at baseline, prior to any functionalization, by generating transfer curves from which the Dirac point was determined. In Figure 7a, a representative bare graphene transfer curve is shown, in which the source-drain current is plotted against the gate voltage applied to the reference electrode, exhibiting the characteristic minimum at the Dirac point (0.309 V).

To assess the stability of the GFET sensors and the noise levels of the measurement setup, repeat Dirac point measurements were taken over 45 min on six sensors in a single GFET chip under constant conditions. Figure 7b shows the variation in the minima associated with the Dirac point for each of the six working sensors as a function of time. The drift in the Dirac point was less than 10 mV, which can be considered moderate depending on the surface density. For example, for a proof-of-concept using the densities of ~10^12^ molecules/cm^2^ that we measured with our functionalization protocol, the expected peak-to-peak potential that we obtain from linear scaling our calculations is approximately 40 mV. Importantly, the observed Dirac point drift is monotonic and largely linear over the measurement window. This behavior is advantageous for downstream signal processing as linear drift components can be suppressed in the second derivative analyses. Overall, these results establish a sufficiently stable electrical baseline for resolving the charge transitions expected from the immobilized amino acids during pH titration.

### 5.5. Evaluating the pH Sensitivity of GFET Sensors

**Figure 8:**
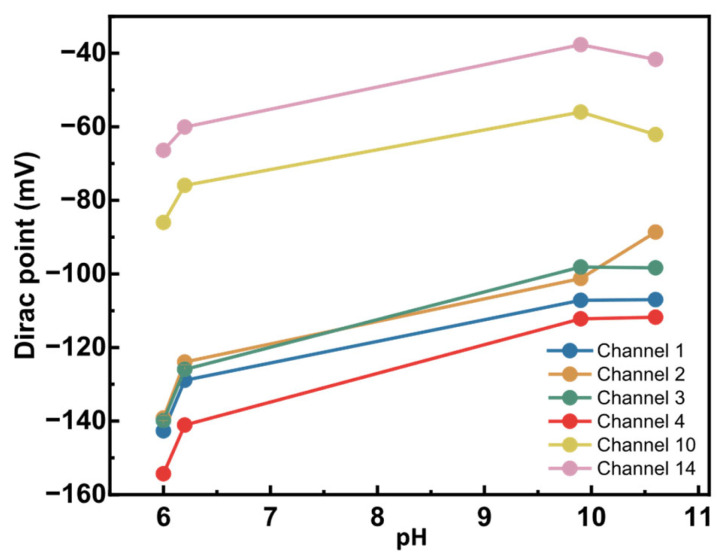
Alkaline pH sensitivity of bare graphene sensors. Six sensors from a single graphene chip were titrated from pH 6 to 10.6. The Dirac point was measured using an external Ag/AgCl reference electrode and was plotted at each defined pH.

To assess any pH sensitivity, we titrated bare graphene sensors. For this initial test, a few pH values from 6 to 10.6 were explored by manual titration. KNO_3_ (neutral) and KOH (alkaline) buffers were chosen to maintain constant ionic strength as the pH is changed. Figure 8 shows the changes in the Dirac points of six working sensors as pH increased.

A measurable pH response was observed, with the Dirac point shifting by 28 mV to 50 mV over a 4.6 pH unit range. Sensors started showing very high resistance and were lost at pH > 10.6. Consequently, this prevented measurements in more alkaline conditions. The observed pH sensitivity in the graphene sensors can be due to several factors. First, chemical vapor deposition (CVD)-grown graphene often contains defects and grain boundaries, which can be oxidized to introduce oxygen- and hydrogen-containing functional groups [34]. These moieties can be protonated or deprotonated depending on pH, thereby altering the local charge density and shifting the Dirac point. Second, alkaline solutions, such as KOH, could penetrate the SiO_2_ passivation layer, creating diffusion pathways for ions that alter the electrostatic environment near graphene. Third, the SiO_2_ substrate itself holds charges and ions that, with the application of a voltage bias, can be charged or discharged and influence graphene’s electronic properties.

Since the observed residual pH response arises from certain surface- or substrate-related functional groups, their pKa values will be distinct from the amino acids. The second derivative analysis provides a means of separating these contributions as it enhances the inflection points associated with amino acid protonation and deprotonation. Hence, while Figure 8 suggests a pH-dependent shift in the Dirac point, contributions from other factors must also be considered when interpreting the observed pH sensitivity of the graphene sensors. This pH response of bare graphene establishes a necessary baseline for interpreting amino acid-specific electrochemical signatures in future measurements.

Full integration of the SPR-validated surface chemistry with robust electrochemical readout is ongoing; once completed, this combined approach will enable direct correlation between immobilized molecules and the resulting GFET signals, providing a quantitative framework for amino acid fingerprinting.

## 6. Conclusions

In this article, we reviewed the most promising emerging next-generation protein sequencing technologies, including nanopore-based approaches, amino acid-to-DNA barcoding, fluorescence-based sequencing on semiconductor chips, microfluidic Edman degradation, and base-induced N-terminal cleavage chemistry. Across these different methods, a common theme emerges: while significant progress has been made in controlling peptide processing and readout, direct, label-free identification of individual amino acids remains unresolved.

Nanopore-based approaches have shown notable advances, and, in our view, they are the closest to commercialization owing to the established frameworks adapted from nucleotide sequencing. However, reliable discrimination among all 20 standard amino acids typically requires engineered pores, carrier peptides, or signal deconvolution. Similarly, converting amino acids into DNA readouts leverages mature NGS platforms used for genomics but require iterative binding and labeling cycles for accurate amino acid identification, resulting in long, complex workflows. Fluorescence-based approaches achieve high throughput but fundamentally encode sparse fingerprints or classify amino acids into groups based on physicochemical similarities, necessitating database matching for sequence inference rather than residue-level identification.

Recent chemical approaches to amino acid cleavage have advanced beyond classical Edman chemistry, promising efficient protein manipulation for more compact systems. In particular, a recently developed alkaline-based cleavage chemistry preserves the native charge and amphoteric behavior of amino acids, providing an opportunity to access intrinsic residue properties. Nevertheless, identification in these workflows still relies on MS or fluorescence readouts rather than direct sensing of the amino acids themselves.

To tackle this gap, the platform that we propose in this work has been designed to resolve the intrinsic pH-dependent electrochemical fingerprints of amino acids as a direct way of residue identification. Calculations show that the amphoteric properties of amino acids induce measurable changes in surface potential, with a peak-to-peak signal amplitude of approximately 0.4 mV per 100 molecules/μm^2^, scaling with molecular surface coverage. Graphene, with its exceptional sensitivity to small changes in surface potential, lack of parasitic functional groups, and large surface area, is an ideal material for detecting these electrochemical signatures.

Here, we establish the experimental foundations for amino acid electrochemical profiling using GFETs. We optimized a graphene functionalization protocol using PMA as linker to achieve directional amino acid immobilization via amide bond formation. Surface densities reached ~3 × 10^12^ molecules/cm^2^, quantified using SPR. Based on the calculated surface potential model and experimentally achieved surface coverages, the expected GFET response may extend to approximately 40 mV. Additionally, we have shown that the system allows in situ synthesis of peptides on graphene up to three amino acids in length using solid-phase peptide chemistry, enabling further tuning of the surface’s amphoteric properties by adding charged amino acids.

To host these measurements, we developed three custom sensor-microfluidic platforms for: (i) preconditioning of the GFET chips for baseline uniformity, (ii) parallel functionalization of up to three amino acids on a single chip, and (iii) controlled pH titration and stable electrochemical measurements. GFET characterization showed well-defined Dirac points, with drift measuring less than 10 mV over 45 min. Amino acid fingerprints are expected to emerge from the second derivative analysis of the transfer curves, which suppresses linear drift and noise contribution while enhancing the amphoteric charge transitions. This suggests a promising signal-to-noise ratio for resolving amino acid electrochemical signatures.

Residual pH sensitivity ranged from 28 to 50 mV over a 4.6 pH range. Further titration experiments are needed to determine its origin—potentially from the underlying SiO_2_ substrate or ion permeation through graphene. Such residual responses are expected to exhibit distinct pKa signatures, allowing them to be characterized separately from the amino acid fingerprints during analysis.

Together, these results establish a graphene-based platform capable of directly probing amino acid electrochemical fingerprints—a key element currently missing in emerging next-generation protein sequencing technologies. Future work will focus on determining the limits of amino acid discrimination and on benchmarking the system’s performance.

As seen now and moving forward, artificial intelligence and machine learning will play central roles in enabling whole-protein and single-point amino acid sequencing. Similar to nucleic acid sequencing, next-generation protein sequencing is unlikely to rely on a single universal approach; instead, a combination of complementary techniques will be needed to sequence the proteome completely. In this context, GFET-based sensing offers a label-free, scalable approach that directly accesses intrinsic amino acid properties and can integrate emerging chemical cleavage and peptide-processing strategies.

## Figures and Tables

**Table 1:** Theoretical values for the $d_{A}$ and *dn*/*dc* of the adsorbed molecules.

Molecule	Molecular Volume (Å^3^)/Surface Area (Å^2^)	$d_{A}$(Å)	*dn*/*dc* (cm^3^/g)
**1-pyrenemethylamine**	26 Å^2^	5.099	0.094 * [22]
**glutamic acid**	140.2 Å^3^ [23]	5.195	0.183 [24]
**lysine**	170.3 Å^3^ [23]	5.543	0.181 [24]
**aspartic acid**	113.1 Å^3^ [23]	4.836	0.197 [24]

* Substituted with analogous values where data was unavailable.

## Data Availability

All datasets generated during the study are available upon request from the corresponding author.

## Supplementary Materials

The following supporting information can be downloaded at: https://www.mdpi.com/article/10.3390/bios16020083/s1, Figure S1: Calculated surface potential (Ψ) of 100 molecules/μm^2^ of C-terminal–immobilized Lys, Lys–Asp, and Lys–Asp–Lys on a field-effect transistor (FET) as a function of pH. The addition of residues and charged sidechains modulate the surface potential, resulting in distinct pH-dependent responses for each peptide; Figure S2: Comparison of antibody recognition for peptide sequence DYKGGG synthesized with and without intermediate acetylation steps. DKKGGG serves as control sequence as the mutation at the second amino acid position should prevent antibody recognition. SPR binding kinetics assay show that the peptides synthesized without acetylation (left) exhibit a significantly higher nonspecific antibody response, whereas incorporation of acetylation steps effectively suppresses undesired interactions.

## Abbreviations

The following abbreviations are used in this manuscript:

α-HL	α-hemolysin
ATZ	Anilinothiazolinone
BCS	Barcode cycle sequencing
CB7	Cucurbit{7}uril
ClpX	Caseinolytic protease X
CsgG	Curlin sigma S-dependent growth subunit G
CVD	Chemical vapor deposition
DDT	1-Dodecanethiol
DIPEA	N,N-diisopropylethylamine
DMSO	Dimethylsulfoxide
FET	Field-effect transistor
FFKM	Perfluoroelastomer
GFET	Graphene field-effect transistor
HBTU	Hexafluorophosphate benzotriazole tetramethyl uranium
MALDI-TOF	Matrix-assisted laser desorption/ionization-time-of-flight
MS	Mass spectrometry
NGS	Next-generation sequencing
PBA	1-Pyrenebutyric acid
PBASE	1-Pyrenebutyric acid N-hydroxysuccinimide ester
PBS	Phosphate-buffered saline
PCB	Printed circuit board
PDMS	Polydimethylsiloxane
PEEK	Polyether ether ketone
PITC	Phenylisothiocyanate
PMA	1-Pyrenemethylamine
POC	Peptide-oligonucleotide conjugate
PTC	Phenylthiocarbamyl
PTH	Phenylthiohydantoin
PTM	Post-translational modification
QCM-D	Quartz crystal microbalance with dissipation
RI	Refractive index
SPR	Surface plasmon resonance
TFA	Trifluoroacetic acid
TIRF	Total internal reflection fluorescence
ZCP	Zero crossover point
