# Supplementary material for: A Graphene Field-Effect Transistor-Based Biosensor Platform for the Electrochemical Profiling of Amino Acids
Source: Biosensors (Basel). 2026 Jan 29;16(2):83. doi: 10.3390/bios16020083 (PMC12938010; doi:10.3390/bios16020083)
Supplement: Supplementary file 1 [file biosensors-16-00083-s001.zip › biosensors-4076358-supplementary.pdf]

biosensors

MDPI

Article

A Graphene Field-Effect Transistor-Based Biosensor Platform for the Electrochemical Profiling of Amino Acids

Roanne Deanne Aves [1,2], Janwa El-Maiss [1], Divya Balakrishnan [1], Naveen Kumar [3], Mafalda Abrantes [4], Jérôme Borme [4], Vihar Georgiev [3], Pedro Alpuim [4,5], César Pascual García [1,*]

[1] Luxembourg Institute of Science and Technology (LIST), 28 avenue des Hauts-Fourneaux, L-4362, Esch-sur-Alzette, Luxembourg
[2] University of Luxembourg, 2 place de l'Université, L-4365 Esch-sur-Alzette, Luxembourg
[3] University of Glasgow, University Avenue, Glasgow G12 8QQ, United Kingdom
[4] International Iberian Nanotechnology Laboratory (INL), Avenida Mestre José Veiga s/n, 4715-330 Braga, Portugal
[5] Center of Physics of the Universities of Minho and Porto, University of Minho, 4710-057, Braga, Portugal

* Correspondence: cesar.pascual@list.lu

Supplementary Material

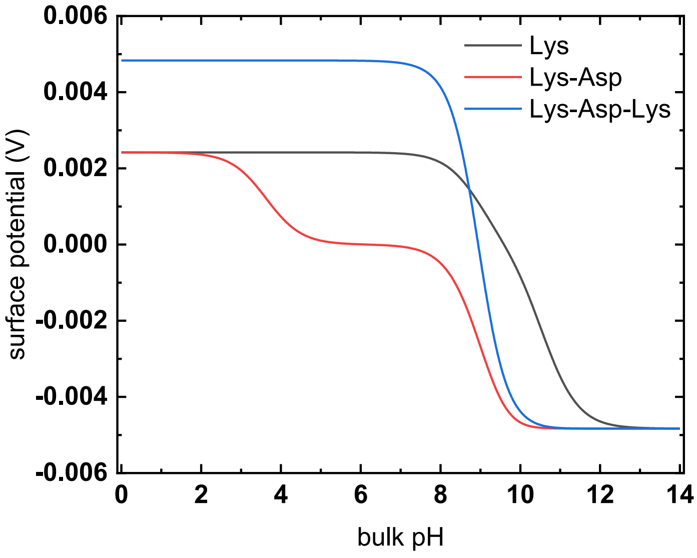

Figure S1. Calculated surface potential (Ψ) of 100 molecules/μm² of C-terminal–immobilized Lys, Lys–Asp, and Lys–Asp–Lys on a field-effect transistor (FET) as a function of pH. The addition of residues and charged sidechains modulate the surface potential, resulting in distinct pH-dependent responses for each peptide.

Received: date
Revised: date
Accepted: date
Published: date

Citation: To be added by editorial staff during production.

Copyright: © 2025 by the authors. Submitted for possible open access publication under the terms and conditions of the Creative Commons Attribution (CC BY) license (https://creativecommons.org/licenses/by/4.0/).

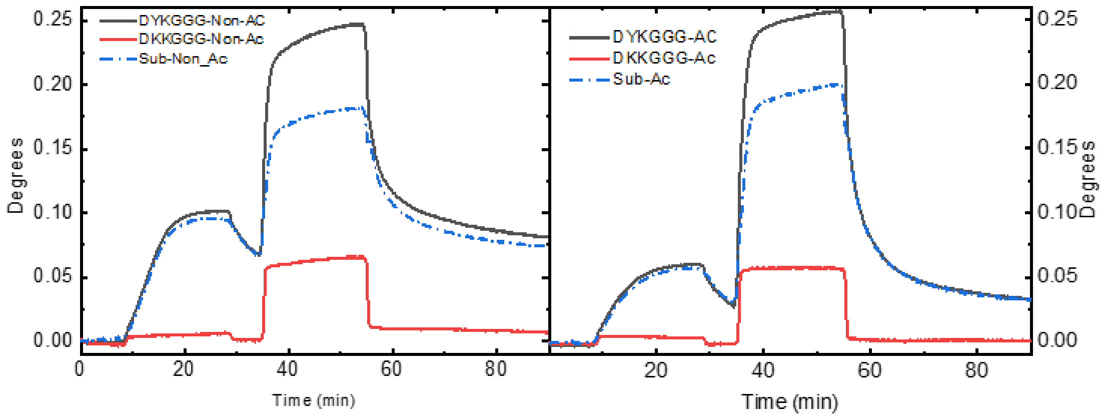

Figure S2. Comparison of antibody recognition for peptide sequence DYKGGG synthesized with and without intermediate acetylation steps. DKKGGG serves as control sequence as the mutation at the second amino acid position should prevent antibody recognition. SPR binding kinetics assay show that the peptides synthesized without acetylation (left) exhibit a significantly higher nonspecific antibody response, whereas incorporation of acetylation steps effectively suppresses undesired interactions.

To validate the significance of acetylation during solid-phase peptide synthesis, the peptide sequence DYKGGG, which has a corresponding antibody, was synthesized either with or without acetylation steps following each coupling reaction. Antibody recognition was subsequently assessed through SPR binding kinetics assays. While the acetylation reactions produced SPR angle shifts below the reliable quantification threshold due to the introduction of few, small molecules, their impact on downstream antibody binding was clearly observable. The peptide synthesized without acetylation resulted in higher nonspecific antibody response, consistent with the presence of unreacted amino groups leading to uncontrolled chain elongation and a heterogeneously functionalized surface. In contrast, peptides synthesized with intermediate acetylation exhibited improved specificity, confirming the effective passivation of residual reactive sites.
